# Potential of MALDI-TOF-based serum *N*-glycan analysis for the diagnosis and surveillance of breast cancer

**DOI:** 10.1038/s41598-020-76195-y

**Published:** 2020-11-05

**Authors:** Jong Won Lee, Kyungsoo Lee, Sei Hyun Ahn, Byung Ho Son, Beom Seok Ko, Hee Jeong Kim, Il Yong Chung, Jisun Kim, Woochang Lee, Myung-Su Ko, Soojeong Choi, Suhwan Chang, Chung Kon Ko, Sae Byul Lee, Dong-Chan Kim

**Affiliations:** 1 Division of Breast Surgery, Department of Surgery, Asan Medical Center, University of Ulsan College of Medicine, 88, Olympic-ro 43-gil, Songpa-gu, Seoul, 05505 Republic of Korea; 2 R&D Center, NOSQUEST Inc., 660, Daewangpangyo-ro, Bundang-gu, Seongnam-si, Gyeonggi-do 13494 Republic of Korea; 3 Department of Laboratory Medicine, Asan Medical Center, University of Ulsan College of Medicine, Seoul, Republic of Korea; 4 Health Screening and Promotion Center, Asan Medical Center, Seoul, Republic of Korea; 5 Department of Biomedical Sciences, Asan Medical Center, University of Ulsan College of Medicine, Seoul, Republic of Korea

**Keywords:** Breast cancer, Cancer

## Abstract

Matrix-assisted laser desorption/ionization time-of-flight mass spectrometry (MALDI-TOF MS)-based serum *N*-glycan analysis has gained acknowledgment for the diagnosis of breast cancer in recent years. In this study, the possibilities of expanding its application for breast cancer management and surveillance were discovered and evaluated. First, a novel MALDI-TOF platform, IDsys RT, was confirmed to be effective for breast cancer analysis, showing a maximum area under the curve of 0.91. Multiple *N*-glycan markers were identified and validated using this process, and they were found to be applicable for differentiating recurring breast cancer samples from healthy control or ordinary breast cancer samples. Recurrence samples were especially distinct from non-recurrence samples when *N*-glycan signatures were sampled in multiple time points and monitored via MALDI-TOF, throughout the therapy. These results suggested the feasibility of MALDI-TOF-based *N*-glycan analysis for tracking the molecular signatures of breast cancer and predicting recurrence.

## Introduction

Breast cancer is the most common cancer in women worldwide, exceeding 6 million estimated cases in 2018^1^. In 2016, the average breast cancer death rate per 100,000 women reached 20% in the United States^2^. In practice, breast cancer is frequently diagnosed by various methods including biopsy, mammography, ultrasonography, and magnetic resonance imaging (MRI)^3–5^. However, these methods are usually invasive and time-consuming. Alternatively, molecule-based and relatively non-invasive strategies have been suggested, and have made their way to practical usage. These include the analysis of tumor-indicating proteins or nucleic acids within the patient’s body fluid^6,7^. However, despite the simplicity of the procedures and their feasibility in terms of scale-down, the methods have frequently shown low accuracy, and are, therefore, too limited to be a fully independent prognostic indicator. Sensitivity and specificity of CA 15-3, one of the FDA-approved protein biomarkers, had reached only up to 65.7 and 76.7%, respectively^8^. While the low serum levels of protein markers have hindered their reliability in the early stages of breast cancer, efforts have been made to predict metastasis by monitoring the levels of CA15-3^9^. However, weak correlation between CA15-3 and prognosis remains a challenge for its universal use^9,10^.

In recent years, alterations in protein glycosylation have been introduced as promising biomarkers for cancer diagnosis. Accordingly, serum *N*-glycan analysis has been successfully utilized in various diseases including breast cancer^11,12^. Furthermore, the ability to incorporate a fast, price-competitive, and high-throughput platform, such as matrix-assisted laser desorption/ionization time-of-flight mass spectrometry (MALDI-TOF MS), greatly helps the impact of this application^13–15^. Analysis of a mass spectrum can provide a deeper insight into a disease by simultaneous analysis of multiple biomarkers^16^. Evidence of the relationship between *N*-glycan expression and breast cancer prognosis has also been reported in recent years, suggesting the potential of *N*-glycans as prognostic markers^17^. Previously, we had only demonstrated the classification capacity of MALDI-TOF by comparing breast cancer and healthy control samples^14^. In this study, we aimed to explore the possibility of expanding the application of MALDI-TOF-based *N*-glycan analysis to breast cancer diagnosis by obtaining recurrence samples and samples acquired during follow-up.

## Results

### N-Glycan signatures in patients with breast cancer

**Figure 1:**
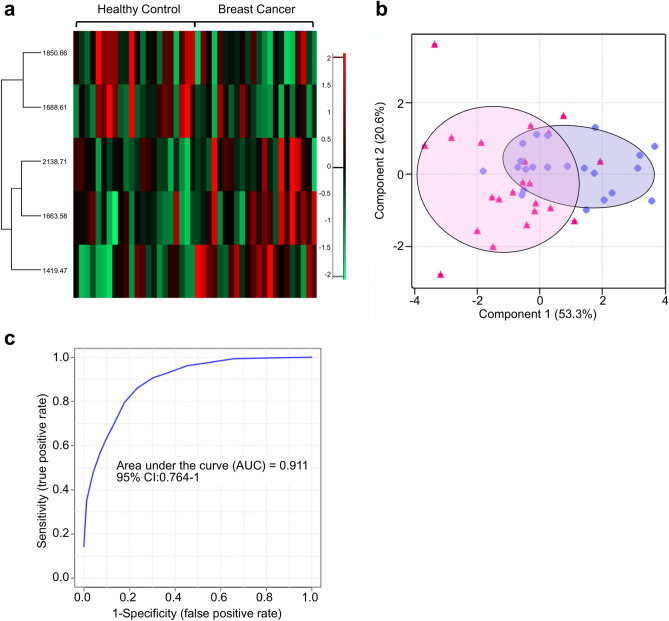
*N*-Glycan intensity comparison of healthy controls and breast cancer samples. (**a**) Heat map showing normalized intensity profiles of representative *N*-glycans for comparing healthy controls and breast cancer samples. (**b**) PCA plot and (**c**) ROC curve for healthy controls (closed triangle) and breast cancer samples (closed circle).

First, the ability of MALDI-TOF to identify healthy and breast cancer samples was evaluated. For this evaluation, 22 healthy control samples and an equal number of breast cancer samples were subjected to *N*-glycan purification and MALDI-TOF analysis. At least 2 samples from every breast cancer stage (stage 0 to IV) were included, in order to incorporate various characteristics into the sample group. Furthermore, in order to eliminate the bias arising from patient age in relation to the *N*-glycan profile, the average age of the healthy control and breast cancer groups was balanced to 53.7 and 51.5 years, respectively. As shown in Fig. 1a, five *N*-glycan markers (*m/z* 1419, 1663, 1688, 1850, and 2138) were significant (*P-*value < 0.05) in distinguishing between healthy and breast cancer samples. Heatmap visualization revealed that three out of the five (*m/z* 1419, 1663, and 2138) were positive markers, whose MS intensities increased in breast cancer, whereas the other two (*m/z* 1688 and 1850) were negative markers with an opposite behavior. Likewise, separate clusters of two sample groups were seen in PCA analysis (Fig. 1b), and the maximum AUC value was 0.91 (Fig. 1c). The model trained by support vector machine (SVM) could identify healthy control and breast cancer samples with an accuracy of 88.6% (Supplementary Fig. S1). The intensity difference of the selected markers was also clearly demonstrated by comparing the average with overall *N*-glycan intensities exceeding 90% of data frequency (Supplementary Fig. S2). Additionally, raw spectrum profiles of the selected marker and their *N*-glycan structures are shown in Supplementary Fig. S3. In order to validate the quantitative analysis of IDsys RT and reliability of selected biomarkers, *N*-glycan preparation and MALDI-TOF analysis of samples were conducted together with a standard serum in the same batch. The experimental configuration for the standard sera is shown in Supplementary Fig. S4. One standard serum sample was added for every 11 experimental samples, yielding a total of 16 standard spectral data. Since the intensities were normalized based on total ion currents (TIC), relative standard deviation (RSD) of the normalized intensities of standard sera were plotted for *N*-glycan *m/z* exceeding 90% of data frequency (Supplementary Fig. S5A). Most of the glycan intensities were shown to have low RSD values of < 0.2. Moreover, quantitative calibration curves between the TIC value and five individual selected marker intensities were plotted (Supplementary Fig. S5B–F). While the RSD value of the intensities was the lowest (0.075) in the middle *m/z *range (*m/z* 1622) of the spectrum, glycans with higher *m/z* values tended to be relatively poorly quantified.

### N-Glycan signatures in patients with recurring breast cancer

**Figure 2:**
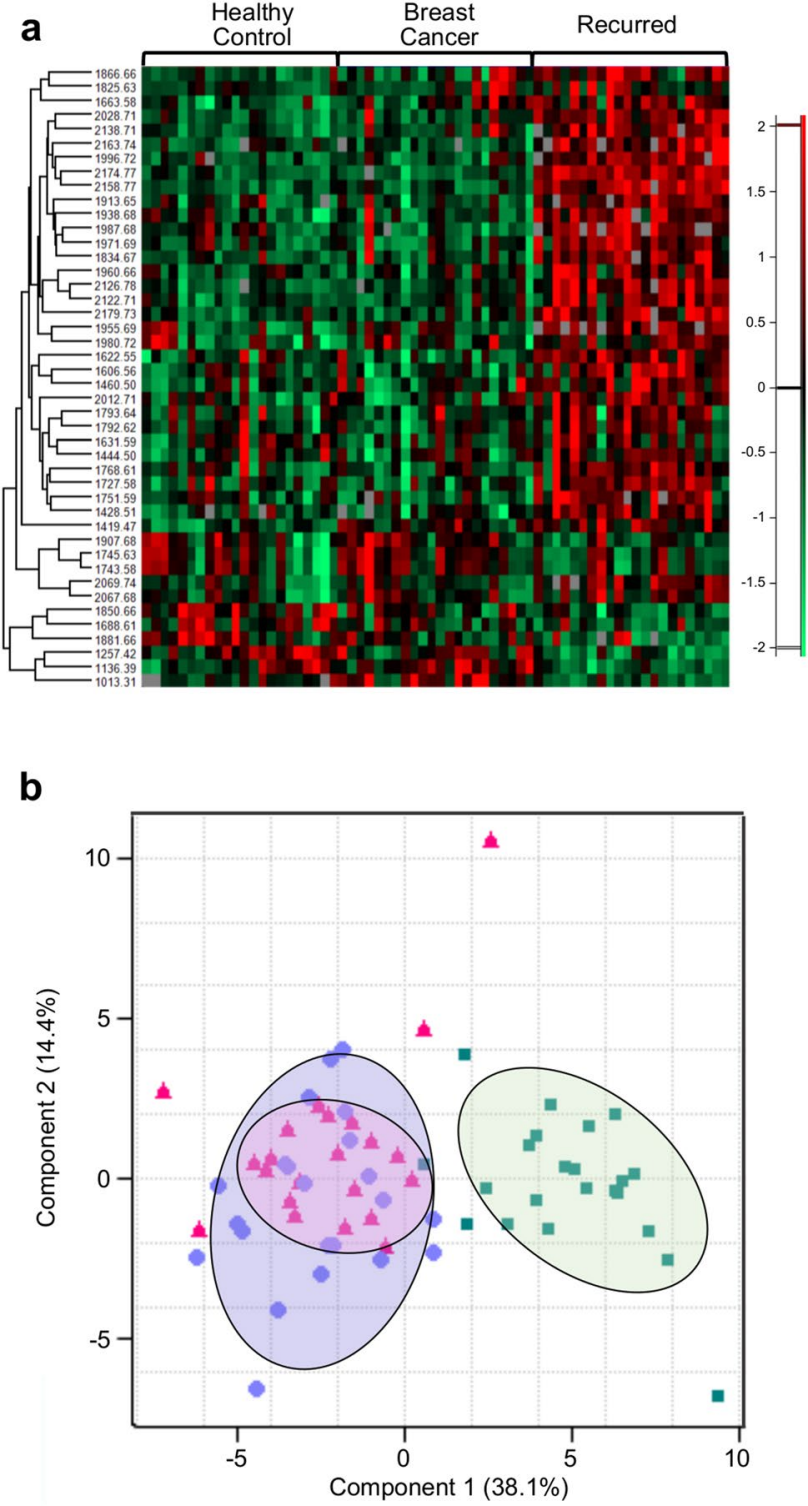
*N*-Glycan intensity comparison of healthy controls, breast cancer, and recurrence samples. (**a**) Heatmap showing the intensity profiles of representative *N*-glycans comparing healthy controls, breast cancer, and recurrence samples. (**b**) PCA plot for healthy controls (closed triangle), breast cancer (closed circle), and recurrence samples (closed square).

Additional analyses were performed by comparing the sera of 22 patients with recurring breast cancer with those from healthy control and breast cancer groups. Recurring samples included various types of recurrence, namely local, regional, distant, and their combinations. The average age was 47.5 years Addition of recurring samples in the analysis led to a distinct separation of *N*-glycan signatures, with a significantly increased number of *N*-glycans (a total of 41) (Fig. 2a). When comparing the combined healthy and breast cancer samples with recurrence samples, the markers seemed to be divided into positive and negative. Interestingly, all the positive and negative markers that were found to correspond to breast cancer, in the previous section, were included in the 41 glycans and showed the same tendency towards recurring samples. This tendency was clearly visible when the intensity profile of all five candidate markers in all three experimental groups were compared (Supplementary Fig. S6A–E). When visualized by PCA, the cluster of recurring samples was clearly separated from the healthy control and breast cancer samples (Fig. 2b). In addition, despite the significant difference between healthy controls and breast cancer samples in the previous result, the difference was less compared to that with recurring samples.

### Monitoring N-glycan reaction to breast cancer therapy

In order to evaluate the feasibility of expanding the application of MALDI-TOF MS-based *N*-glycan profile analysis towards breast cancer surveillance, the *N*-glycan profile shift in breast cancer therapy was monitored. Sera were collected thrice from 22 patients with breast cancer, throughout the therapy, i.e., before, during, and after treatment. Among the 22 patients, 11 ended up being diagnosed with recurrence while the rest did not show evidence of any recurrence. For the group with 11 cured, at least 2 patients, initially diagnosed with each breast cancer stage (stage 0 to III), were included in order to incorporate various characteristics into the sample group, and the average age was 43.3 years. These samples were compared with the 22 healthy control samples. The median follow-up (f/u) time for monitoring the group of patients with breast cancer was 64.5 months. As shown in Supplementary Fig. S7A, samples of patients under therapy showed a distinctive *N*-glycan profile compared to those of healthy controls. However, although the patients were defined to be cured, the post-treatment sample signature generally resembled that of the pre- and mid-treatment samples rather than that of healthy control samples. Similarly, in the PCA plot, healthy control samples formed a clearly separated cluster while the pre-, mid-, and post-treatment samples could not be distinguished from each other (Supplementary Fig. S7B).

### Exploring N-glycan markers for predicting breast cancer recurrence

**Figure 3:**
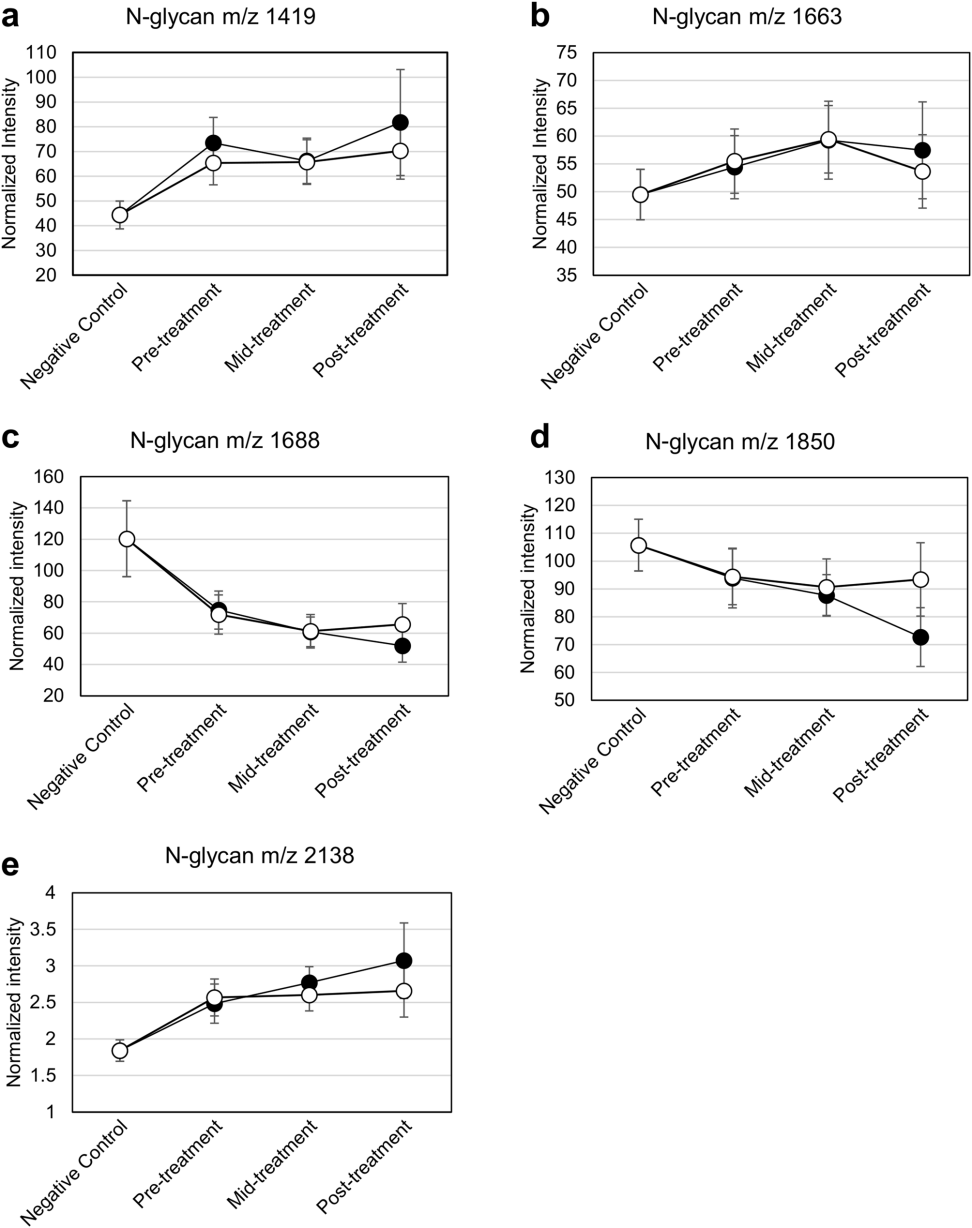
Comparison of the *N*-glycans’ [(**a**) M + Na 1419, (**b**) M + Na 1663, (**c**) M + Na 1688, (**d**) M + Na 1850 and (**e**) M + Na 2138] intensity profile of NED (open circle) and recurrence samples (closed circle).

To gain a deeper insight into the *N*-glycan shift under the influence of therapy, changes in the average intensities were monitored and plotted for the 5 *N*-glycan markers (*m/z* 1419, 1663, 1688, 1850, and 2138) that were dominant in healthy samples than in breast cancer. The group of 11 patients, whose cancer recurred after treatment, was monitored in the same manner and results compared to that of the cured group. Based on this comparison, we sought to validate the *N*-glycan markers’ ability to track or predict recurrence. The recurring samples in this group included patients initially diagnosed with various breast cancer stages and various types of recurrence. The average age was 44.9 years. A heatmap comparing the recurrence patients with healthy controls showed a similar result as seen in that with patients with No Evidence of Disease (NED) (Supplementary Fig. S8). The criteria for a prediction marker included the following: first, pre-treatment samples should show different (either increased or decreased) intensities compared to healthy controls; second, the changed intensity should be restored towards healthy controls only in NED samples, not in recurrence samples. All pre-treatment samples showed shifted *N*-glycan intensities compared to healthy samples, as expected (Fig. 3A–E). In addition, the intensity patterns were mostly similar in the NED and recurrence sample groups until the mid-treatment time-point. However, only the post-treatment samples from patients with NED showed a tendency to restore or maintain *N*-glycan intensity as in healthy controls. Rather than profiling the five marker candidates for NED and recurred samples, additional analysis was performed to describe the statistically relevant markers for discriminating NED from recurred samples. Results revealed a set of four glycans (M + Na 1565, 1727, 1881, and 2012) (Supplementary Fig. S9), in which the five original glycan marker candidates were not included.

## Discussion

Prior to discussing the feasibility of expanding MALDI-TOF-based *N*-glycan analysis to breast cancer diagnosis, the diagnostic performance of the system needed to be addressed, especially because no previous study had reported breast cancer diagnosis using IDsys RT. In this study, 22 samples of healthy controls and 22 breast cancer samples were successfully discriminated, with a maximum AUC value of 0.91, indicating IDsys RT to be applicable as a valuable tool for breast cancer analysis. The initially identified positive (*m/z* 1419, 1663, and 2138) and negative markers (*m/z* 1688, 1850) showed consistency in follow-up experiments. Furthermore, this result was in good agreement with previous literature concerning *N*-glycan biomarkers in breast cancer. *N*-glycan *m/z* 1419, a high-mannose type glycan (M6N2), has been frequently brought to attention in several studies using MALDI-TOF^16,18,19^. Furthermore, *N*-glycan *m/z* 1663 was reported to be upregulated when carcinoma tissues were imaged using MALDI-TOF^20,21^.

The *N*-glycan profile of recurrence samples has been found to show drastic differences relative to healthy controls and breast cancer samples. Importantly, all the 5 *N*-glycan markers, identified earlier, remained significant in the recurrence samples; the positive breast cancer markers tended to increase further in recurrence, whereas the negative markers decreased further. This gave us the insight that the *N*-glycan breast cancer markers can also be applied for predicting recurrence. Moreover, the markers showed significantly different intensities when NED samples and recurrence samples were compared, which further supported the validity of glycans as surveillance markers. Post-treatment signatures of NED samples were closer to those of pre- and mid-treatment samples than of healthy controls. Despite the unintuitive result, one might consider an exposure to extensive cancer therapy to possibly lead to multivariate shifts in molecular expression profiles and require a longer follow-up period for complete restoration. Ideally, a time-tracking study of a single patient would lead to a more accurate conclusion, rather than a group-based comparison across different individuals.

Indeed, although *N*-glycan markers showed differential patterns between NED and recurrence, the wide range of error still remains an issue. Due to the limited number of samples used in this study, expanded research would be required in the future to elucidate a more accurate definition of surveillance markers and reach a practical application. Furthermore, the original set of five glycan marker candidates did not share any common glycan species with the set obtained by statistical analysis of NED and recurred samples. Moreover, the four *N*-glycans in the latter set lacked any evidence of differential expression in breast cancer. However, it should be noted that recurrence samples used in this study included patients with various types of recurrence, not only local recurrences but also regional and distant ones, which might have led to a distinct expression of glycan pattern in recurred samples than in ordinary breast cancer samples. While it has been claimed that metastasis can significantly affect serum *N*-glycans, strict control of variables would also be required in further studies.

In conclusion, we have demonstrated the application of MALDI-TOF-based *N*-glycan profile analysis in breast cancer and its recurrence. Using *N*-glycan biomarkers, we suggested a multi-marker-based approach to be viable in terms of characterizing and discriminating specific states of breast cancer, such as recurrence or ongoing therapy. Despite the limited number and conditions used in this study, we believe the *N*-glycan analysis showed promising potential in the management and surveillance of breast cancer. Collective and additional efforts using larger cohorts would provide a more accurate and deeper insight for its practical application.

## Methods

### Subjects and blood collection

The study was approved by the review board of Asan Medical Center (AMC; Seoul, South Korea, IRB approval number: 2018-1234). Blood was collected from both patients with cancer and healthy volunteers at AMC. All study procedures were in accordance with the relevant guidelines, as per the Declaration of Helsinki. Written informed consent was obtained from all the participants involved in the study. Patient characteristics used in this study were collected from AMC and are shown in Table 1 and Supplementary Table S1. For samples assigned to the group named ‘Monitoring’, blood was collected thrice, i.e., before, during, and after breast cancer treatment, yielding 3 samples per patient. For serum preparation, blood was centrifuged at 1000 × *g* for 10 min at 25 °C after a short incubation at 25 °C. Separated serum was stored at − 80 °C in 1.5 mL microcentrifuge tubes (Eppendorf, Hamburg, Germany). Human serum (Sigma-Aldrich, St. Louis, MO, USA) was purchased and applied as standard samples for quantitative validation.

**Table 1:** Information on the samples used in this study.

Categories	Stage	Recurrence type	Sample count	Avg. age	Total count
Healthy control	–	–	22	53.7	22
Breast cancer	0	–	5	51.5	22
	I	–	5		
	II	–	4		
	III	–	6		
	IV	–	2		
Recurring	–	Local	6	47.5	22
		Regional	5		
		Distant	8		
		Local and regional	1		
		Regional and distant	2		
**Monitoring**					
Treated and no evidence of disease (NED)	0	–	6	43.3	33
	I	–	6		
	II	–	12		
	III	–	9		
Treated and recurring	0	Local	3	44.9	33
	I	Local	3		
		Regional	3		
		Distant	3		
		Local and distant	3		
	II	Regional	3		
		Distant	6		
	III	Distant	9		

### N-Glycan preparation

*N*-Glycan preparation, including enzymatic deglycosylation and purification by solid phase extraction (SPE), was performed based on previously described methods^14^. Serum *N*-glycans were released by an enzymatic reaction using PNGase F, followed by a purification step using columns packed with porous graphite carbon (PGC). For *N*-glycan elution, 20% acetonitrile (ACN)/water (v/v) was used in all cases.

### Analytical methods

Sample and matrix preparation for MALDI-TOF MS analysis was conducted as described previously^14^. For preparing the matrix solution, 2,5-dihydroxybenzoic acid (DHB) was dissolved in a mixture of 40 mM sodium chloride solution and ACN (3:1, v/v) to a final concentration of 20 mg/mL. Purified N-glycan solution was mixed with the matrix solution with a volume ratio of 1:2, and 1 μL of the mixture was spotted onto MALDI plate and vacuum dried for analysis. In MALDI-TOF MS analysis, mass spectra were acquired using IDsys RT MALDI-TOF MS (ASTA, Suwon-si, South Korea). Positive ion reflection mode was used and a mass range of 900 to 3000 Daltons was analyzed. Only *N*-glycan mass peaks above 2 S/N (signal-to-noise) ratio were considered as valid peaks. Mass calibration was performed for every analyte using the IDsys RT built-in post-processing steps, with respect to 13 reference theoretical ‘known-glycan’ masses (M + Na 1257, 1419, 1485, 1501, 1542, 1581, 1647, 1688, 1704, 1743, 1809, 1850, and 1905, decimals not shown here) with an error acceptance of 100 ppm.

### Data processing

Peak information from the mass spectrum was extracted using the software IDsys 3.0 (ASTA, Suwon-si, South Korea). Among all the obtained peaks, *m/z* features corresponding to a reference *N*-glycan list consisting of 156 human glycans were filtered and used; the list was modified from previous literature^22^. The reference *N*-glycans are listed in Supplementary Table S2. Absolute peak intensity (API_*i*_) of each *N*-glycan was normalized by the sum of all APIs, yielding the normalized absolute peak intensity (NAPI_*i*_) using a previously described formula^14^.

### Visualization and statistical analysis

Processed feature matrices were analyzed using Perseus™ 1.5.2.6 (Max Planck Institute of Biochemistry, Berlin, Germany). The *m/z* features were filtered by a *P-*value cutoff of 0.05 resulting from a multiple-sample ANOVA test. For hierarchical clustering and visualization, all data were *Z*-score normalized. Similarly, principle component analysis (PCA) and its visualization were performed. Receiver operating characteristic (ROC) analysis and corresponding area under the curve (AUC) values were obtained using the biomarker analysis tool in MetaboAnalystR, a public web-based biological data analysis package^23^. Linear SVM was used as the classifying method.

## Supplementary information

Supplementary Information.
