# Supplementary material for: Potential of MALDI-TOF-based serum N-glycan analysis for the diagnosis and surveillance of breast cancer
Source: Sci Rep. 2020 Nov 5;10:19136. doi: 10.1038/s41598-020-76195-y (PMC7644762; doi:10.1038/s41598-020-76195-y)
Supplement: Supplementary file 1 — Supplementary Information. [file 41598_2020_76195_MOESM1_ESM.docx]

**Potential of MALDI-TOF-based serum *N*-glycan analysis for the diagnosis and surveillance of breast cancer**

Jong Won Lee^1†^, Kyungsoo Lee^2†^, Sei Hyun Ahn^1^, Byung Ho Son^1^, Beom Seok Ko^1^, Hee Jeong Kim^1^, Il Yong Chung^1^, Jisun Kim^1^, Woochang Lee^3^, Myung-Su Ko^4^, Soojeong Choi^1^, Suhwan Chang^5^, Chung Kon Ko^2^, Sae Byul Lee^1*^, Dong-Chan Kim^2*^

^1^ Division of Breast Surgery, Department of Surgery, Asan Medical Center, University of Ulsan College of Medicine, Seoul, Republic of Korea

### ^2^ R&D Center, NOSQUEST Inc., Seongnam, Gyeonggi, Republic of Korea

^3^ Department of Laboratory Medicine, Asan Medical Center, University of Ulsan College of Medicine, Seoul, Republic of Korea

^4^ Health Screening and Promotion Center, Asan Medical Center, Seoul, Republic of Korea

^5^ Department of Biomedical Sciences, Asan Medical Center, University of Ulsan College of Medicine, Seoul, Republic of Korea

### ^†^These authors contributed equally.

***Corresponding authors**:

Sae Byul Lee

Division of Breast Surgery, Department of Surgery, Asan Medical Center, University of Ulsan College of Medicine, 88, Olympic-ro 43-gil, Songpa-gu, Seoul 05505, Republic of Korea

Phone: +82-10-7209-4620; Fax: +82-2-3010-6710

Email: newstar153@hanmail.net

Dong-Chan Kim

R&D Center, NOSQUEST Inc., 660, Daewangpangyo-ro, Bundang-gu, Seongnam-si, Gyeonggi-do 13494, Republic of Korea

Tel: +82-10-4527-3736; Fax: +82-31-606-1855

Email: jenokin@nate.com

**Supplementary Tables**

**Supplementary Table S1**. Basic characteristics of the study population

|  | **Breast cancer group** | | **Recurred group** | | **Monitoring group** | |
| --- | --- | --- | --- | --- | --- | --- |
|  | **(n =22)** | | **(n =22)** | | **(n =22)** | |
| Age at diagnosis  (years, Mean±SD) | 51.5±8.8 | | 47.5±11.0 | | 43.3±5.1 | |
|  | count | Percentage (%) | count | Percentage (%) | count | Percentage (%) |
| **T stage** |  |  |  |  |  |  |
| T0/Tis | 5 | 22.7 | 1 | 4.5 | 0 | 0.0 |
| T1 | 4 | 18.2 | 12 | 54.5 | 0 | 0.0 |
| T2 | 8 | 36.4 | 7 | 31.8 | 12 | 54.5 |
| T3 | 3 | 13.6 | 2 | 9.1 | 10 | 45.5 |
| T4 | 1 | 4.5 | 0 | 0.0 | 0 | 0.0 |
| unknown | 1 | 4.5 | 0 | 0.0 | 0 | 0.0 |
| **N stage** |  |  |  |  |  |  |
| N0 | 7 | 31.8 | 8 | 36.4 | 7 | 31.8 |
| N1 | 8 | 36.4 | 8 | 36.4 | 9 | 40.9 |
| N2 | 1 | 4.5 | 1 | 4.5 | 1 | 2.0 |
| N3 | 5 | 22.7 | 5 | 22.7 | 5 | 22.7 |
| unknown | 1 | 4.5 | 0 | 0.0 | 0 | 0.0 |
| **Stage** |  |  |  |  |  |  |
| 0 | 0 | 0.0 | 1 | 4.5 | 0 | 0.0 |
| I | 7 | 31.8 | 7 | 31.8 | 0 | 0.0 |
| II | 6 | 27.3 | 7 | 31.8 | 12 | 54.5 |
| III | 7 | 31.8 | 6 | 27.3 | 10 | 45.5 |
| IV | 1 | 4.5 | 1 | 4.5 | 0 | 0.0 |
| unknown | 1 | 4.5 | 0 | 0.0 | 0 | 0.0 |
| **Nuclear grade** |  |  |  |  |  |  |
| I | 2 | 9.1 | 1 | 4.5 | 0 | 0.0 |
| II | 15 | 68.2 | 10 | 45.5 | 14 | 63.6 |
| III | 4 | 18.2 | 7 | 31.8 | 8 | 36.4 |
| unknown | 1 | 4.5 | 4 | 18.2 | 0 | 0.0 |
| **Histologic grade** |  |  |  |  |  |  |
| I | 1 | 4.5 | 0 | 0.0 | 0 | 0.0 |
| II | 11 | 50.0 | 10 | 45.5 | 14 | 63.6 |
| III | 5 | 22.7 | 7 | 31.8 | 8 | 36.4 |
| Unknown | 5 | 22.7 | 5 | 22.7 | 0 | 0.0 |
|  |  |  |  |  |  |  |
| Hormonal receptor (+) | 17 | 77.3 | 17 | 77.3 | 14 | 63.6 |
| HER2 (+) | 6 | 27.3 | 3 | 13.6 | 2 | 9.1 |
|  |  |  |  |  |  |  |
| Chemotherapy | 12 | 54.5 | 18 | 81.8 | 22 | 100.0 |
| Hormonal therapy | 16 | 72.7 | 16 | 72.7 | 15 | 68.2 |
| Radiation therapy | 18 | 81.8 | 12 | 54.5 | 15 | 68.2 |
| Targeted therapy | 4 | 18.2 | 2 | 9.1 | 2 | 9.1 |

HER2 = human epidermal growth factor receptor 2

**Supplementary Table S2**. The reference *N*-glycans

| No. | *N*-glycan mass (M, Da) | Monoisotopic mass (M+Na, Da) |
| --- | --- | --- |
| 1 | 910.328011 | 933.31778 |
| 2 | 1056.385919 | 1079.375688 |
| 3 | 1072.380834 | 1095.370603 |
| 4 | 1113.407383 | 1136.397152 |
| 5 | 1218.438743 | 1241.428512 |
| 6 | 1234.433657 | 1257.423426 |
| 7 | 1259.465292 | 1282.455061 |
| 8 | 1275.460207 | 1298.449976 |
| 9 | 1316.486756 | 1339.476525 |
| 10 | 1380.491566 | 1403.481335 |
| 11 | 1396.486481 | 1419.47625 |
| 12 | 1405.523201 | 1428.51297 |
| 13 | 1421.518115 | 1444.507884 |
| 14 | 1437.51303 | 1460.502799 |
| 15 | 1478.539579 | 1501.529348 |
| 16 | 1519.566128 | 1542.555897 |
| 17 | 1542.54439 | 1565.534159 |
| 18 | 1558.539304 | 1581.529073 |
| 19 | 1566.555623 | 1589.545392 |
| 20 | 1567.576024 | 1590.565793 |
| 21 | 1583.570939 | 1606.560708 |
| 22 | 1588.537568 | 1611.527337 |
| 23 | 1599.565853 | 1622.555622 |
| 24 | 1608.602573 | 1631.592342 |
| 25 | 1640.592402 | 1663.582171 |
| 26 | 1665.624037 | 1688.613806 |
| 27 | 1681.618952 | 1704.608721 |
| 28 | 1704.597213 | 1727.586982 |
| 29 | 1712.613532 | 1735.603301 |
| 30 | 1720.592128 | 1743.581897 |
| 31 | 1722.645501 | 1745.63527 |
| 32 | 1728.608446 | 1751.598215 |
| 33 | 1729.628848 | 1752.618617 |
| 34 | 1745.623762 | 1768.613531 |
| 35 | 1750.590392 | 1773.580161 |
| 36 | 1754.660482 | 1777.650251 |
| 37 | 1769.634996 | 1792.624765 |
| 38 | 1770.655397 | 1793.645166 |
| 39 | 1802.645226 | 1825.634995 |
| 40 | 1811.681946 | 1834.671715 |
| 41 | 1827.67686 | 1850.666629 |
| 42 | 1843.671775 | 1866.661544 |
| 43 | 1858.671441 | 1881.66121 |
| 44 | 1866.650037 | 1889.639806 |
| 45 | 1868.703409 | 1891.693178 |
| 46 | 1874.666355 | 1897.656124 |
| 47 | 1882.644951 | 1905.63472 |
| 48 | 1884.698324 | 1907.688093 |
| 49 | 1890.66127 | 1913.651039 |
| 50 | 1891.681671 | 1914.67144 |
| 51 | 1912.643215 | 1935.632984 |
| 52 | 1915.692904 | 1938.682673 |
| 53 | 1916.713305 | 1939.703074 |
| 54 | 1925.724873 | 1948.714642 |
| 55 | 1931.687819 | 1954.677588 |
| 56 | 1932.70822 | 1955.697989 |
| 57 | 1937.67485 | 1960.664619 |
| 58 | 1948.703135 | 1971.692904 |
| 59 | 1953.669764 | 1976.659533 |
| 60 | 1957.739855 | 1980.729624 |
| 61 | 1964.698049 | 1987.687818 |
| 62 | 1972.714368 | 1995.704137 |
| 63 | 1973.734769 | 1996.724538 |
| 64 | 1989.729684 | 2012.719453 |
| 65 | 2005.724598 | 2028.714367 |
| 66 | 2014.761318 | 2037.751087 |
| 67 | 2020.724264 | 2043.714033 |
| 68 | 2028.70286 | 2051.692629 |
| 69 | 2030.756233 | 2053.746002 |
| 70 | 2036.719179 | 2059.708948 |
| 71 | 2044.697775 | 2067.687544 |
| 72 | 2046.751148 | 2069.740917 |
| 73 | 2061.750813 | 2084.740582 |
| 74 | 2071.782782 | 2094.772551 |
| 75 | 2077.745728 | 2100.735497 |
| 76 | 2078.766129 | 2101.755898 |
| 77 | 2083.732758 | 2106.722527 |
| 78 | 2087.777697 | 2110.767466 |
| 79 | 2093.740642 | 2116.730411 |
| 80 | 2094.761044 | 2117.750813 |
| 81 | 2099.727673 | 2122.717442 |
| 82 | 2103.797763 | 2126.787532 |
| 83 | 2110.755958 | 2133.745727 |
| 84 | 2115.722588 | 2138.712357 |
| 85 | 2118.772277 | 2141.762046 |
| 86 | 2119.792678 | 2142.782447 |
| 87 | 2134.767192 | 2157.756961 |
| 88 | 2135.787593 | 2158.777362 |
| 89 | 2140.754222 | 2163.743991 |
| 90 | 2151.782507 | 2174.772276 |
| 91 | 2156.749137 | 2179.738906 |
| 92 | 2160.819227 | 2183.808996 |
| 93 | 2167.777422 | 2190.767191 |
| 94 | 2175.793741 | 2198.78351 |
| 95 | 2176.814142 | 2199.803911 |
| 96 | 2182.777088 | 2205.766857 |
| 97 | 2190.755683 | 2213.745452 |
| 98 | 2192.809056 | 2215.798825 |
| 99 | 2204.759033 | 2227.748802 |
| 100 | 2206.750598 | 2229.740367 |
| 101 | 2207.808722 | 2230.798491 |
| 102 | 2208.803971 | 2231.79374 |
| 103 | 2217.840691 | 2240.83046 |
| 104 | 2222.783236 | 2245.773005 |
| 105 | 2223.803637 | 2246.793406 |
| 106 | 2233.835605 | 2256.825374 |
| 107 | 2239.798551 | 2262.78832 |
| 108 | 2240.818952 | 2263.808721 |
| 109 | 2244.765181 | 2267.75495 |
| 110 | 2245.785582 | 2268.775351 |
| 111 | 2249.83052 | 2272.820289 |
| 112 | 2255.793466 | 2278.783235 |
| 113 | 2256.813867 | 2279.803636 |
| 114 | 2264.830186 | 2287.819955 |
| 115 | 2265.850587 | 2288.840356 |
| 116 | 2280.8251 | 2303.814869 |
| 117 | 2281.845501 | 2304.83527 |
| 118 | 2296.820015 | 2319.809784 |
| 119 | 2297.840416 | 2320.830185 |
| 120 | 2302.807045 | 2325.796814 |
| 121 | 2306.877136 | 2329.866905 |
| 122 | 2313.835331 | 2336.8251 |
| 123 | 2318.80196 | 2341.791729 |
| 124 | 2321.851649 | 2344.841418 |
| 125 | 2322.872051 | 2345.86182 |
| 126 | 2337.846564 | 2360.836333 |
| 127 | 2338.866965 | 2361.856734 |
| 128 | 2352.808507 | 2375.798276 |
| 129 | 2354.86188 | 2377.851649 |
| 130 | 2363.8986 | 2386.888369 |
| 131 | 2368.803421 | 2391.79319 |
| 132 | 2368.841144 | 2391.830913 |
| 133 | 2369.861545 | 2392.851314 |
| 134 | 2370.856794 | 2393.846563 |
| 135 | 2378.873113 | 2401.862882 |
| 136 | 2379.893514 | 2402.883283 |
| 137 | 2384.836059 | 2407.825828 |
| 138 | 2385.85646 | 2408.846229 |
| 139 | 2390.823089 | 2413.812858 |
| 140 | 2395.888429 | 2418.878198 |
| 141 | 2401.851375 | 2424.841144 |
| 142 | 2402.871776 | 2425.861545 |
| 143 | 2410.888095 | 2433.877864 |
| 144 | 2411.883344 | 2434.873113 |
| 145 | 2425.862608 | 2448.852377 |
| 146 | 2426.883009 | 2449.872778 |
| 147 | 2427.90341 | 2450.893179 |
| 148 | 2442.877924 | 2465.867693 |
| 149 | 2443.898325 | 2466.888094 |
| 150 | 2448.864954 | 2471.854723 |
| 151 | 2452.935045 | 2475.924814 |
| 152 | 2458.872838 | 2481.862607 |
| 153 | 2459.893239 | 2482.883008 |
| 154 | 2464.859869 | 2487.849638 |
| 155 | 2467.909558 | 2490.899327 |
| 156 | 2468.929959 | 2491.919728 |

**Supplementary Figures**


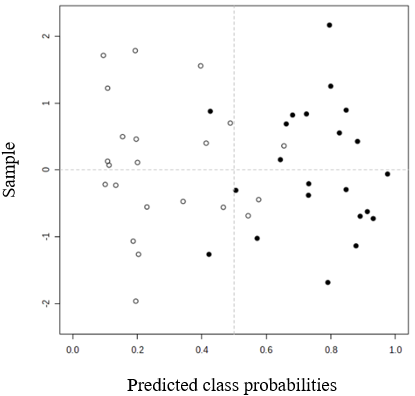


**Supplementary Figure S1**. The predicted class probabilities (average of the cross-validation) for each sample from healthy control (open circle, ○) and breast cancer (closed circle,●) groups using the classifier selected in ROC analysis.

**Supplementary Figure S2**. Comparison of the average processed spectra of healthy controls (blue bars) and patients with breast cancer (red bars). Results are shown as the mean value ± standard deviation (SD) of 22 samples in each group.


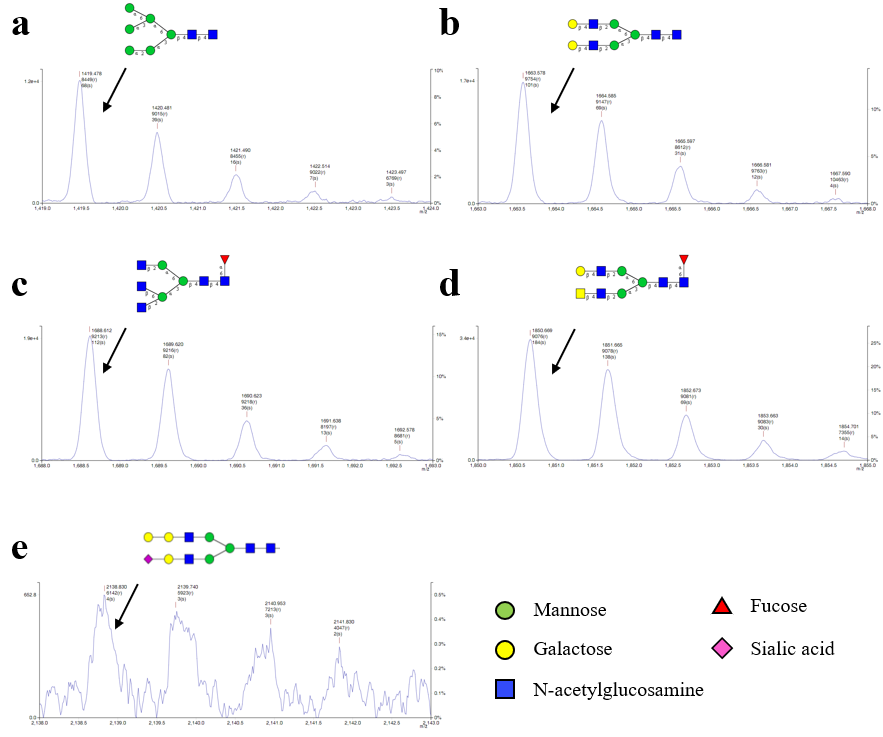


**Supplementary Figure S3**. Zoomed-in spectral images of five glycan marker candidates (**[a]**M+Na 1419, **[b]**M+Na 1663, **[c]**M+Na 1688, **[d]** M+Na 1850, and **[e]**M+Na 2138) obtained from the mass spectrum of a randomly-selected breast cancer patient. Arrows indicate the monoisotopic mass peaks.


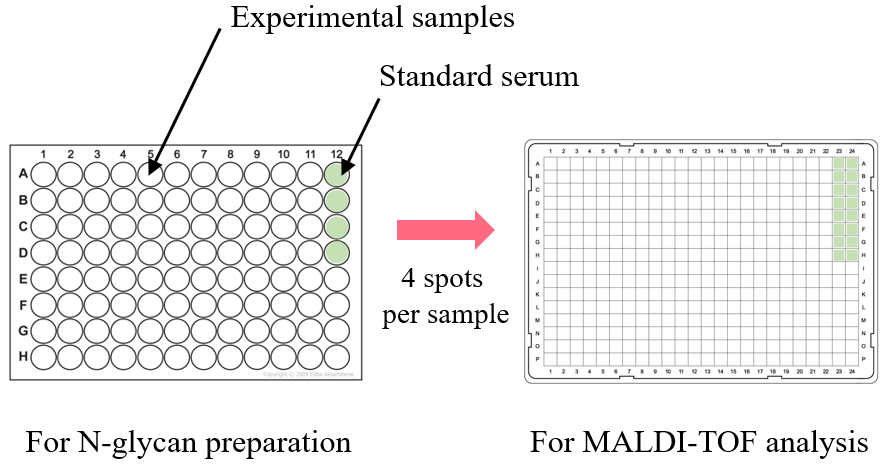


**Supplementary Figure S4**. Representation of the configuration of standard serum sample usage in quantitative validation.


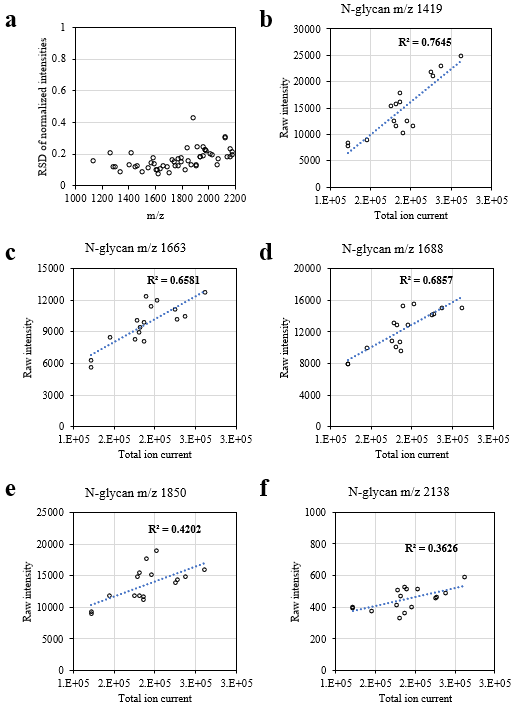


**Supplementary Figure S5**. Results of the quantitative validation of IDsys RT using standard serum. **[a]** RSD values of normalized glycan intensities plotted with respect to glycan m/z values. Each spot represents individual glycan species. Calibration curves plotted using raw intensities of glycans (**[b]**M+Na 1419, **[c]**M+Na 1663, **[d]**M+Na 1688, **[e]**M+Na 1850, and **[f]**M+Na 2138) versus TIC.


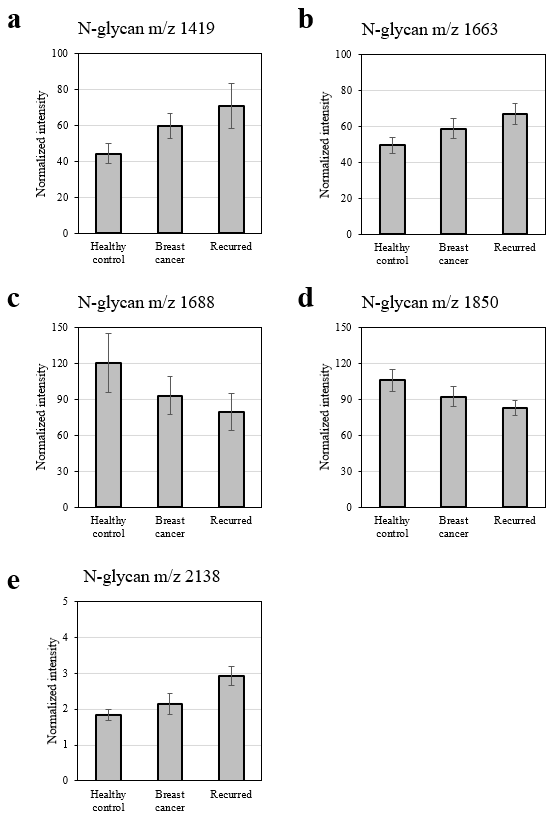


**Supplementary Figure S6**. Comparison of normalized intensities of glycans (**[a]**M+Na 1419, **[b]**M+Na 1663, **[c]**M+Na 1688, **[d]**M+Na 1850, and **[e]**M+Na 2138) for healthy control, breast cancer, and recurred sample groups. Results are shown as the mean value ± standard deviation (SD) of 22 samples in each group.


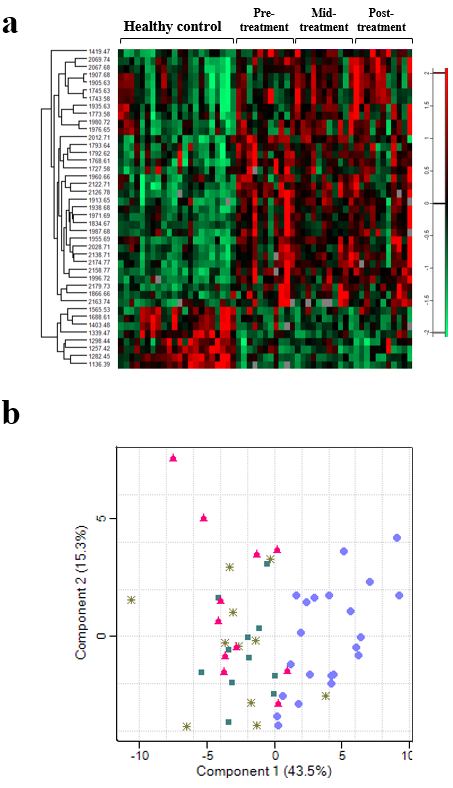


**Supplementary Figure S7**. Comparison of *N*-glycan intensity across healthy controls, pre-, mid-, and post-treatment samples (NED after treatment). **(a)** Heatmap showing the intensity profiles of representative *N*-glycans.**(b)** PCA plot for healthy controls (closed circle,●), pre-treatment (closed triangle,▲), mid-treatment (closed square, ■), and post-treatment (star, *).


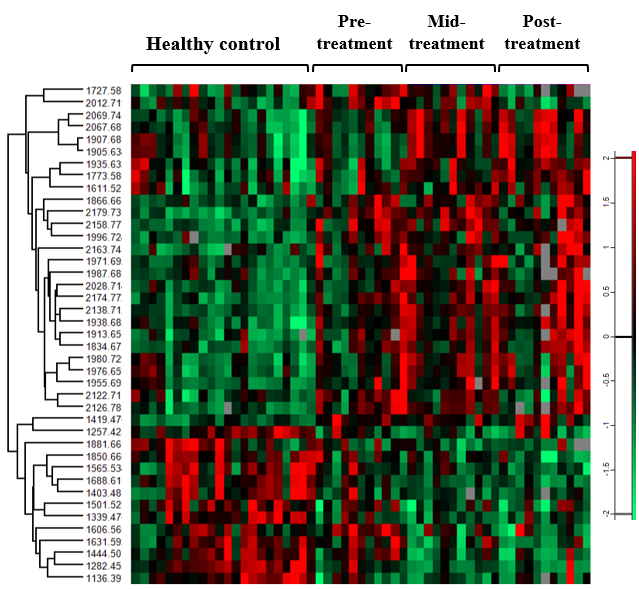


**Supplementary Figure S8**. Heatmap showing *N*-glycan intensity profiles of healthy controls, pre-, mid-, and post-treatment samples (recurring after treatment).


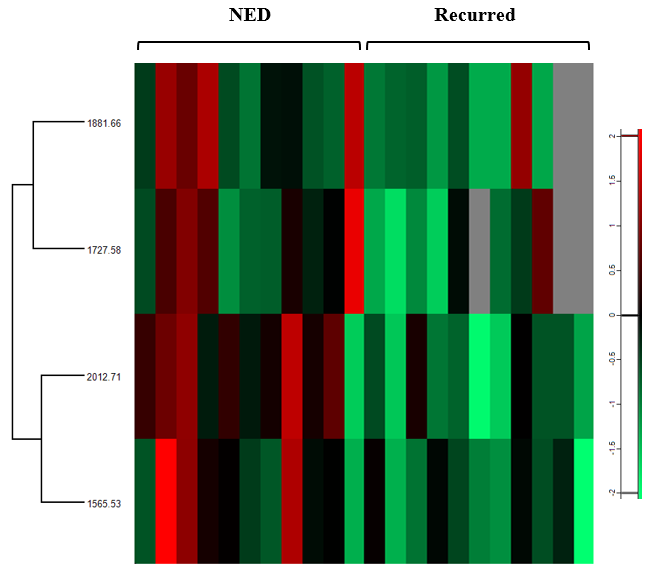


**Supplementary Figure S9**. Heatmap showing *N*-glycan intensity profiles of the post-treatment samples in NED and recurred sample groups.
